# Beneficial Role of ROS in Cell Survival: Moderate Increases in H_2_O_2_ Production Induced by Hepatocyte Isolation Mediate Stress Adaptation and Enhanced Survival

**DOI:** 10.3390/antiox8100434

**Published:** 2019-10-01

**Authors:** Izak Patrik Miller, Ivan Pavlović, Borut Poljšak, Dušan Šuput, Irina Milisav

**Affiliations:** 1 Faculty of Medicine, Institute of Pathophysiology, University of Ljubljana, Zaloska 4, SI-1000 Ljubljana, Slovenia; negotium.personale@gmail.com (I.P.M.); dusan.suput@mf.uni-lj.si (D.Š.); 2 Laboratory of Molecular Biology and Endocrinology, Vinca Institute of Nuclear Sciences, University of Belgrade, Mike Petrovica Alasa 12-14, P.O. Box 522, 11001 Belgrade, Serbia; pavlovic@vin.bg.ac.rs; 3 Laboratory of Oxidative Stress Research, Faculty of Health Sciences, University of Ljubljana, Zdravstvena pot 5, SI-1000 Ljubljana, Slovenia; borut.poljsak@zf.uni-lj.si

**Keywords:** hydrogen peroxide, mitochondria, redox regulation, caspase-9, apoptosis

## Abstract

High levels of reactive oxygen species (ROS) can lead to impairment of cell structure, biomolecules’ loss of function and cell death and are associated with liver diseases. Cells that survive increased ROS often undergo malignant transformation. Many cancer cells tolerate high levels of ROS. Here we report a transiently increased production of H_2_O_2_ and concomitant upregulation of antioxidative enzymes triggered by hepatocyte isolation; the H_2_O_2_ levels revert in about two days in culture. Three-day survival rate of the isolated cells in the presence of 2.5-fold increase of H_2_O_2_ is almost 80%. Apoptosis activation through the mitochondrial pathway is meanwhile reduced by inhibition of caspase-9 triggering. This reduction depends on the amount of H_2_O_2_ production, as decreased production of H_2_O_2_ in the presence of an antioxidant results in increased apoptosis triggering. These stress adaptations do not influence urea production, which is unchanged throughout the normal and stress adapted phases. We conclude that hepatocytes’ stress adaptation is mediated by increased ROS production. In this case, high ROS improve cell survival.

## 1. Introduction

Reactive oxygen species (ROS) are derivatives of partially reduced molecular oxygen that are formed and degraded by all multicellular aerobic organisms during metabolism [1]. Moderate concentrations of ROS are required for normal cell function, while excessive or too small amounts of ROS lead to oxidative imbalance, named oxidative stress or antioxidative/reductive stress, respectively [2]. ROS include superoxide anion (O2•^−^) and hydroxyl radical (•OH) that are free radicals with an unpaired electron. Non-radical molecules, including hydrogen peroxide (H_2_O_2_), ozone (O_3_) and peroxynitrite (ONOO^−^) are also ROS and can participate in free radical reactions [3]. The most abundant mitochondrial ROS is superoxide. H_2_O_2_ is the main redox sensing, signaling and redox regulation molecule, the oxidant of low pKa cysteine residues in redox-sensitive proteins [4]. H_2_O_2_ interactions can modulate the activity of mammalian transcription factors including AP-1, NRF2, CREB, HSF1, HIF-1, TP53, NF-κB [5]. Redox reactions control many processes in the organism. These include apoptosis, autophagy, aging, circadian rhythm, hypoxia, inflammation, muscle contraction, proliferation, stem cell self-renewal, wound healing and tumorigenesis [4]. ROS can be neutralized by antioxidative enzymes, such as superoxide dismutases (SODs) that catalyze superoxide anion conversion to less reactive H_2_O_2_, to be further reduced to water by glutathione peroxidases (GPx) and catalase (CAT) [4,6]. The two main sources of ROS in hepatocytes are mitochondria and nicotinamide adenine dinucleotide phosphate (NADPH) oxidase [7]. 

Deregulation of ROS is associated with many pathologies; increased oxidative stress is associated with metabolic, cardiovascular, inflammatory and neurodegenerative diseases [1]. Oxidative stress was also detected in liver diseases, including non-alcoholic fatty liver disease, alcoholic liver disease, hepatitis C virus infection and genetic hemochromatosis [6,8]. Increased production of ROS following ischaemia/reperfusion triggers hepatocyte cell death [7,9,10]. Sustained low-grade oxidative stress could promote liver fibrosis and may be associated with increased risk of mid-term graft failure [11]. ROS mediate liver damage during the potential ischemia/reperfusion injury associated with transplant surgery or the tissue microenvironment because of the chronic hepatic inflammation or infection [7].

Various insults, from outside and within the cells, including dysregulation of ROS, can induce apoptosis in hepatocytes [12]. Hepatocyte apoptosis can result from damaged macromolecules and as a consequence of significant alterations of signal transduction pathways [13]. Intracellular stress conditions lead to generation of pro-survival and pro-apoptosis signals that are integrated at mitochondria [14]. Domination of pro-apoptotic signals promotes mitochondrial outer membrane permeabilization, dissipation of mitochondrial transmembrane potential and release of intermembrane space proteins, like cytochrome c, into cytosol, which binds to the cytosolic protein APAF1 to form apoptosome and results in caspase-9 and apoptosis activation through the intrinsic pathway. Extrinsic apoptosis pathway is induced by extracellular stress signals and is propagated through receptors, e.g., FAS/CD95 [14], resulting in caspase-8 activation. In the type II cells, like hepatocytes, caspase-8 signals require amplification through the mitochondrial pathway for caspase-3 activation [14]. Caspase-9 participates in apoptosis initiation through the extrinsic signaling in type II cells, however, there are conflicting reports whether it is essential for caspase-8 initiated apoptosis. Caspase-3 was activated through the mitochondrial pathway from the extracellular trigger in Apaf-1-deficient Jurkat cells without the apoptosome-mediated caspase-9 activation [15]. However, reconstitution of caspase-9 expression restored Fas signaling in caspase-9 deficient T cell line demonstrating the necessity of caspase-9 for the apoptosis triggering [16]. In hepatocytes, mitochondrial pathway of caspase activation is regulated by two anti-apoptotic BCL-2 family proteins, Bcl-xL and Mcl-1 that are interdependently required to maintain hepatocyte integrity [17]. Moderate amounts of stress, unable to result in cell death, can induce stress responses including enhanced defense, repair and cross-resistance to multiple stressors [18,19]. Preapoptotic cell stress response (PACOS) is a stress adaptation of primary hepatocytes that reduces apoptosis initiation through inactivation of caspase-9 triggering [20,21]. Redox regulation is an important mechanism for regulation of stress responses [10,19]. 

Here we report that increased levels of H_2_O_2_ can be a part of normal response to moderate stress in primary hepatocytes. The consequence of this stress includes decreased apoptotic triggering through the mitochondrial pathway. Both adaptations, the increased amount of ROS production and lower apoptosis triggering (PACOS), revert to levels similar to those in liver after some days in culture. The function of hepatocytes is preserved at all times; both in stress adapted and in normal cells. Reduced H_2_O_2_ production by addition of N-acetylcysteine (NAC) abolishes the inhibition of apoptosis triggering; therefore, the beneficial hepatocytes’ stress adaptation is mediated by increased ROS production.

## 2. Materials and Methods 

Chemicals were purchased from Sigma (Taufkirchen, Germany) or Merck (Darmstadt, Germany), unless stated otherwise.

### 2.1. Cell Culturing

Liver and primary hepatocytes were isolated from adult male rats, ethical code number is U34401-44/2014/8, issued by Administration of the Republic of Slovenia for Food Safety, Veterinary Sector and Plant Protection (Wistar-Hannover, Ljubljana, Slovenia, 180–280 g). Reverse two-step perfusion with collagenase was used to isolate primary hepatocytes, as described by Milisav et al. [22]. Hepatocyte viability was at least 90% as determined by Trypan blue exclusion. The cells were seeded on the collagen type I coated surface at density 10^5^ cells/cm^2^ either in Petri dishes (diameter 35 mm, urea production), in flasks (antioxidant enzymes activities) or in 96-well plates (hydrogen peroxide generation). Hepatocytes were first incubated in a humidified atmosphere with 95% air and 5% CO_2_ at 37 °C for four hours in Williams medium E supplemented with 10% fetal bovine serum, penicillin and streptomycin (100 U/mL each) and insulin (0.1 U/mL). Hepatocytes were further cultured in Williams medium E with 0.03% bovine serum albumin, penicillin and streptomycin (50 U/mL each), insulin (0.1 U/mL) and 1 µM hydrocortisone hemisuccinate. When indicated, 25 mM of buffered NAC was added to the perfusion solution and all media during isolation and up to four hours after isolation. Liver were cut in 80 µm slices and were processed as previously described [20]. Each experiment was performed either on primary hepatocytes, liver or liver slices from at least three independent isolations. The material was either snap frozen right after isolation or collected at later time points: 1, 2, 3, 6 or 7 days after the isolation.

Protein concentrations were determined by BCA Protein Assay Kit and Pierce 660 nm Protein Assay (Pierce, Thermo Scientific, Rockford, IL, USA) for detection of caspase, catalase, glutathione peroxidase and superoxide dismutase activities, hydrogen peroxide generation, glutathione concentration and urea production.

### 2.2. Hydrogen Peroxide Detection 

The H_2_O_2_ measurements were performed by Amplex Red Hydrogen Peroxide/Peroxidase Assay Kit as is described by supplier (Molecular Probes, Eugene, OR, USA). Generated H_2_O_2_ was detected upon isolation and on days 3 and 6 after isolation by measuring the change in absorbance caused by released H_2_O_2_ reacting with the Amplex Red reagent. 

### 2.3. Biochemical Analyses 

Total glutathione concentration, SOD and GPx activities were determined by Glutathione, Superoxide Dismutase and Glutathione Peroxidase Assay Kits, respectively, in accordance to the manufacturers’ protocols (Cayman Chemical, Ann Arbor, MI, USA). The measured enzyme activities values were normalized to mg of protein.

CAT activity was measured by decreased concentration of added H_2_O_2_ due to the enzymatic conversion into oxygen and water [23]. The protocol used is based on the original protocol by Maehly and Chance [24]. No interference between CAT and GPx under the assay conditions were reported [24,25]. 

To determine the caspase activation, primary hepatocytes and precision cut liver slices were treated for 3 h with 1 µM staurosporine (STS), which induces apoptosis via the mitochondrial pathway. STS was added to freshly isolated hepatocytes 15 min after isolation. The activities of caspases-3 and -9 were detected by Caspase-Glo 3/7 and Caspase-Glo 9 Assays, respectively (Promega, Madison, WI, USA) according to supplier’s protocols.

A portion of cell lysates in CCLR buffer (Promega, Madison, WI, USA) was pooled and used for immunoblotting. Whole cell lysates in reducing Laemmli-buffer (0.25 M Tris pH 6.8, 8% SDS, 40% glycerol, 0.03% bromophenol blue) were denatured at 95 °C for 5 min and 15 μg of a sample was applied on 14% (*w/v*) acrylamide gels and separated by standard sodium dodecyl sulfate–polyacrylamide gel electrophoresis (SDS-PAGE), blotted onto PVDF membrane (Immobilon-P, Merck-Millipore, Darmstadt, Germany) according to standard procedures. Bcl-xL (ab2568) and Mcl-1 (ab53709) antibodies were purchased from Abcam, Cambridge, UK. The signal was detected by luminescence through the secondary goat anti-rabbit antibodies conjugated to horse radish peroxidase (Bio-Rad, Hercules, CA, USA), visualized using X-ray film and quantified by densitometry using Image Studio Lite software (LI-COR, Lincoln, USA). 

Primary hepatocytes were exposed to 3 mM ammonium chloride in phenol-red-free Williams medium E (supplemented as is described above). Ammonium chloride was added to freshly obtained hepatocytes 15 min after isolation. The medium was harvested every 30 min for 2 h, then the urea concentration was measured as described by Castell and Gomez-Lechon [26]. 

### 2.4. Statistical Analyses

The data obtained from at least three independent experiments were expressed as arithmetic means ± standard deviations (SD) and plotted with Sigma Plot 11.0 (Systat Software, San Jose, CA, USA). Statistical analyses of data were performed with Statistical Package for the Social Sciences, v. 25.0 (SPSS, Chicago, IL, USA). One- and two-way analysis of variance (ANOVA) or Kruskal–Wallis rank sum test (for equal or unequal variances, respectively) was used to compare the multiple sample groups. Post hoc analyses were performed by Bonferroni or Dunnett T3 post hoc tests. The values of comparisons were considered as statistically significant when *P* < 0.05.

## 3. Results

### 3.1. Increase of Hydrogen Peroxide during Hepatocyte Isolation

**Figure 1:**
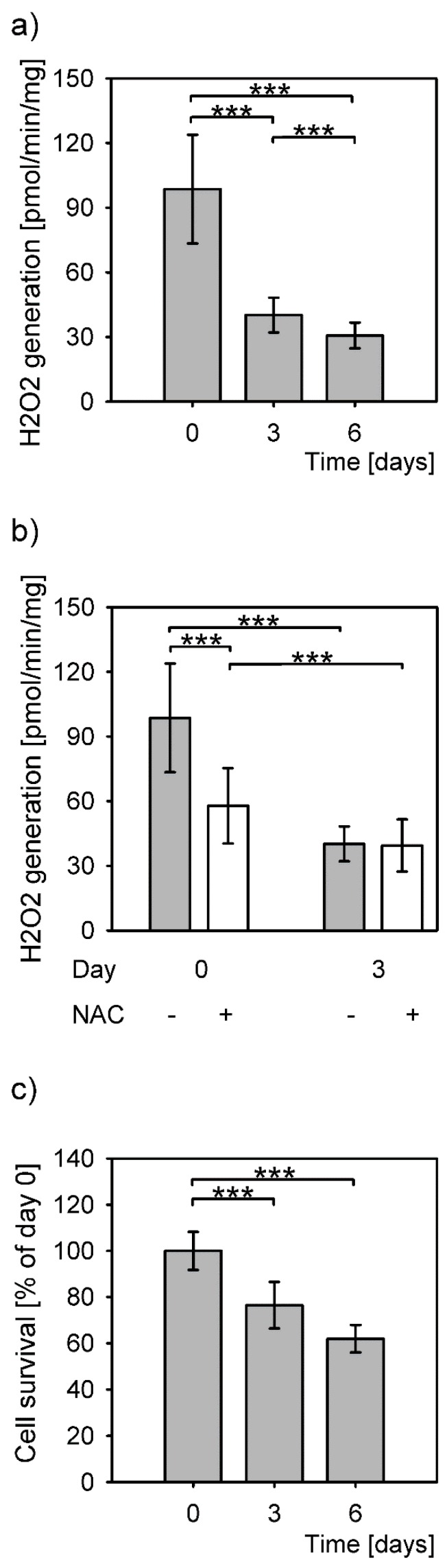
H_2_O_2_ levels and survival of primary hepatocytes. Data are expressed as means ± SD. Statistical significance is represented by “*” symbols ***: *P <* 0.001. (**a**) H_2_O_2_ generation in primary hepatocytes significantly differs among all measured time points (*P* = 1 × 10^−15^, Kruskal-Wallis rank sum test; day 0 to day 3: *P* < 10^−18^; day 0 to day 6: *P* = 6 × 10^−16^; day 3 to day 6: 4 × 10^−5^; Dunnett T3 post hoc test). (**b**) Reduced H_2_O_2_ generation in the presence (+) of N-acetylcysteine (NAC) (*P* = 6 × 10^−18^, Kruskal–Wallis rank sum test). (**c**) Survival of the primary hepatocytes in culture measured as total protein concentrations and expressed as percentage of the protein concentration of the cells attached after isolation (*P* = 0.004, one-way ANOVA). Statistical significance between the cells at time point 0 and 3 days and 0 and 6 days (*P* = 0.039 and *P* = 0.004, respectively, Bonferroni post hoc test).

The highest amount of H_2_O_2_ production by hepatocytes in culture was measured upon isolation (Figure 1a, day 0). The levels of produced H_2_O_2_ decreased statistically significantly on days 3 and 6 post isolation, to 41% and 31% of the isolation level, respectively. The levels of generated H_2_O_2_ differed significantly between all time points (*P* = 1 × 10^−15^, Kruskal–Wallis rank sum test). The difference between the levels of detected H_2_O_2_ between 3 and 6 days after isolation was small, but statistically significant. The addition of an antioxidant, NAC, significantly reduced the H_2_O_2_ production only on the day of isolation (Figure 1b).

Most hepatocytes survived a sharp increase of H_2_O_2_ levels at isolation even without the NAC (Figure 1c). Comparison of the protein amounts from adherent cells right after isolation (100%) to the cells grown for 3 and 6 days in culture revealed a decrease to 76.5% and 62%, respectively. The protein amounts between the days 3 and 6 were not significantly different.

### 3.2. Redox Balancing upon Isolation of Primary Hepatocytes

**Figure 2:**
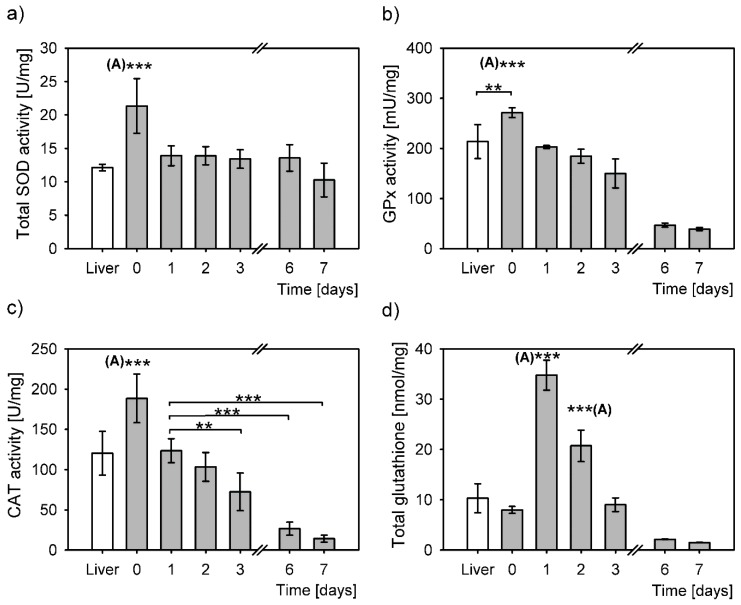
Antioxidative response to oxidative stress in liver (white bars) and in primary hepatocytes at six different time points after cell isolation (grey bars). The values determined in liver and in primary hepatocytes were normalized to mg of total protein and expressed as means ± SD. Statistical significance is represented by “*” symbols (**: *P <* 0.01, ***: *P <* 0.001). “(A)”: significantly differs to all other time points/categories. (**a**) Superoxide dismutase activity (SOD). Overall statistically significant difference between the time points (one-way ANOVA, *P* = 4 × 10^−6^), however only SOD activity right after isolation (day 0) differs significantly to all the other time points (to liver: *P* = 9 × 10^−5^, day 1: *P* = 3 × 10^−4^, day 2: *P* = 2 × 10^−4^, day 3: *P* = 6 × 10^−5^, day 6: *P* = 2 × 10^−4^, day 7: *P* = 1 × 10^−6^, Bonferroni post hoc test). (**b**) Glutathione peroxidase activity (GPx). Overall statistically significant difference between the time points (one-way ANOVA, *P* = 3 × 10^−13^). Bonferroni post hoc test: significant differences between the day 0 cells and the following time points liver: *P* = 0.02, day 1: *P* < 10^−18^, day 2: *P* = 9 × 10^−7^, day 3: *P* = 1 × 10^−8^, day 6: *P* = 3 × 10^−12^, day 7: *P* = 1 × 10^−12^. (**c**) Catalase activity (CAT). The overall difference between the CAT activities in all samples (*P* = 5 × 10^−14^, one-way ANOVA). Bonferroni post hoc test: significant differences between day 0 and the following time points: liver: *P* = 4 × 10^−5^, day 1: *P* = 0.001, day 2: *P* = 2 × 10^−6^, day 3: *P* = 2 × 10^−9^, day 6: *P* = 3 × 10^−13^, day 7: *P* = 1 × 10^−12^. CAT activity in hepatocytes of day 1 compared to day 3: *P* = 0.02, day 6: *P =* 1 × 10^−6^, day 7: *P* = 5 × 10^−7^, Bonferroni post hoc test. (**d**) Total glutathione concentration over days differs significantly (*P =* 1 × 10^−18^, one-way ANOVA). Day 1 to other time points: liver: *P = 1* × 10^−13^, day 0: *P =* 1 × 10^−16^, day 2: *P* = 5 × 10^−10^, day 3: *P* = 2 × 10^−16^, day 6: *P* = 1 × 10^−16^, day 7: *P* = 7 × 10^−17^, Bonferroni post hoc test. Day 2 compared to: liver: *P* = 4 × 10^−6^, day 0: *P* = 2 × 10^−9^, day 3: *P* = 4 × 10^−9^, day 6: *P* = 3 × 10^−11^, day 7: *P* = 2 × 10^−11^, Bonferroni post hoc test.

The cell stress response from isolation, evaluated through measurement of antioxidative enzymes activities and total glutathione concentration, induced a significantly larger antioxidative response compared to that from the snap frozen liver samples (Figure 2). 

SOD activity in isolated hepatocytes cultured from day 1 to day 7 was constant and comparable to that of the liver (Figure 2a). A significant SOD activity increase was observed in hepatocytes right after isolation compared to the liver. This increase is most probably triggered by a sharp increase of O2^•-^ upon hepatocyte isolation. On day 1 post isolation the SOD activity in primary hepatocytes returned to the level of SOD activity in the intact liver, i.e., to roughly two thirds of that observed after isolation and remained unchanged throughout the culturing until day 6, with a slight decrease on day 7. SOD activity differed statistically among the samples (*P* = 4 × 10^−6^, one-way ANOVA), although additional post hoc analyses revealed that it was only SOD activity right after isolation that was significantly different from the rest of the samples. SOD activity on day 0 was at least 1.5-fold higher than on the other days. 

The GPx activity of primary hepatocytes was the highest immediately after isolation (Figure 2b). Compared to the liver, there was 1.3-fold increase in GPx activity in hepatocytes right after isolation; this increase was statistically significant. Gradual statistically significant decreases of GPx activity were observed during culturing in primary hepatocytes. These decreases amounted to 75% on day 1, followed by 68% on day 2 and 55% on day 3 in relation to the levels upon isolation (day 0). The activity of GPx drastically decreased on days 6 and 7 to only 17% and 14% of day 0, respectively. The statistical analysis of glutathione peroxidase (GPx) activity demonstrated an overall significant difference between the samples (*P* = 3 × 10^−13^, one-way ANOVA). The difference between the minimal GPx activity on day 7 and the maximal GPx activity on day 0 was 7-fold. Interestingly, post hoc analyses revealed no difference at all between the GPx activities in the liver and in hepatocytes 1 and 2 days post isolation. The post hoc test revealed no statistically significant differences in GPx activities also between the days 6 and 7.

Overall, CAT activity was significantly different between the samples (*P* = 5 × 10^−14^, one-way ANOVA, Figure 2c). The highest activity in primary hepatocytes was right after isolation and was significantly higher than in the liver and in primary hepatocytes cultured from day 1 to 7. CAT activity returned to the activity level of the liver on day 1 post isolation. CAT activity was steadily declining during the hepatocyte culturing, to reach only 8% of the activity right after isolation on day 7, thus increasing the difference between the maximal and minimal activities to 12-times. Statistically, there was no difference between the CAT activity in liver and in primary hepatocytes on days 1 and 2 post isolation. Compared to day 1, statistically significant decreases of CAT activity were measured in hepatocytes 3, 6 and 7 days post isolation (Figure 2c). 

Total glutathione (GSH/GSSG) concentrations also differed overall (*P* = 1 × 10^−18^, one-way ANOVA). GSH/GSSG right after isolation (day 0) seemed a bit lower; however, its levels did not statistically differ compared to that in the liver. On day 1 post isolation the GSH/GSSG concentration increased by 4.3-fold (compared to day 0) and reached the highest level and significantly differed to that of all the other samples (Figure 2d). GSH/GSSG concentration gradually decreased over time, so that on day 2 it reached 2.6-fold of the levels measured immediately after isolation. This glutathione concentration was also statistically significantly higher from all the other concentrations (Figure 2d). Glutathione levels were restored to the levels in the liver and immediately after the isolation by day 3. Further decreases were measured on days 6 and 7, to a minimum of 19% on day 7. The difference between the highest and the lowest GSH/GSSG concentrations was 22-fold.

### 3.3. Reduced Apoptosis Induction in Stress Adapted Cells

**Figure 3:**
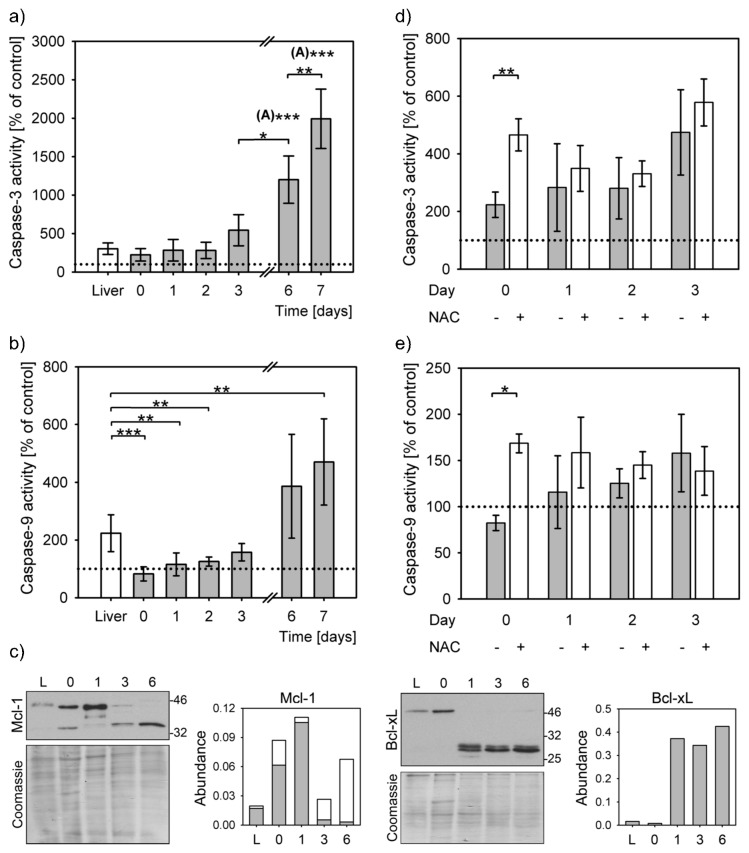
Apoptosis initiation. Caspase activation was induced in staurosporine (STS) treated precision cut liver slices (liver) and hepatocyte cultures from isolation (day 0) to day 7. Bar charts represent caspase activation in STS treated samples normalized to their untreated controls (means ± SD). Caspase activity of untreated controls is indicated by a dotted line. Statistical significance is represented by “*” symbols (*: *P <* 0.05, **: *P <* 0.01, ***: *P <* 0.001). “(A)”: significantly differs to all other time points/categories. (**a**) Activation of caspase-3 in liver slices (white bars) and in primary hepatocytes (grey bars). Caspase-3 activation significantly differs among the STS-treated liver slices and primary hepatocytes (*P =* 1 × 10^−7^, Kruskal–Wallis rank sum test). Apoptosis activation on days 6 and the following time points/categories: liver slices *P =* 0.001; day 0: *P =* 4 × 10^−4^; day 1: *P =* 4 × 10^−4^; day 2: *P =* 4 × 10^−4^; day 3: *P =* 0.027; day 7: *P =* 0.006; Dunnett T3 post hoc test. Apoptosis activation on day 7 and: liver slices *P =* 10^−5^*;* day 0: *P =* 7 × 10^−6^; day 1: *P =* 5 × 10^−6^; day 2: *P =* 4 × 10^−6^; day 3: *P =* 7 × 10^−5^; Dunnett T3 post hoc test. (**b**) Caspase-9 activation in liver slices (white bars) and in primary hepatocytes (grey bars) differs overall among all time points (*P =* 9 × 10^−9^, Kruskal-Wallis rank sum test). The differences between the liver slices and primary hepatocytes at the following time points are: day 0: *P=* 9 × 10^−5^, day 1: *P =* 0.002, day 2: *P =* 0.004, day 7: *P =* 0.014, Dunnett T3 post hoc test. (**c**) Representative immunoblots for Mcl-1 and Bcl-xL. Each lane represents lysates from 3 pooled livers or 3 pooled independent hepatocyte isolations. Densitometric analysis of protein expression normalized to Coomassie stained gels (Abundance), 2–4 repeats. L: liver; 0, 1, 3, 6: days 0, 1, 3, 6, respectively. Mcl-1 quantification: grey bars: full length protein, white bars: short form. (**d**) Caspase-3 activity in the presence (+) and absence (−) of NAC (*P =* 0.003, two-way ANOVA). The difference between untreated and NAC-treated cells on day 0 is statistically significant (*P* = 0.004, Student’s *t*-test). (**e**) Caspase-9 activity in the presence (+) and absence (−) of NAC (*P =* 0.016, two-way ANOVA). The difference between untreated and NAC-treated cells on day 0 is statistically significant (*P* = 0.0003, Student’s *t*-test).

Moderate stress can decrease apoptosis triggering by caspase-9 inactivation (PACOS) [21]. Caspase-3 was activated upon apoptosis induction by staurosporine in precision cut liver slices and primary hepatocytes at all time points. Caspase-3 activities differed significantly overall (*P* = 1 × 10^−7^, Kruskal–Wallis rank sum test). Caspase-3 activity increased by at least 2-fold compared to the untreated controls (Figure 3a). The increase of caspase-3 activity in liver slices was similar to that of the primary hepatocytes at isolation and up to 2 days after that. The caspase-3 activity increased steeply from day 3 and reached the highest level of activation on day 7, i.e., 20-fold more compared to the untreated control, 7-fold to precision cut liver slices and 9-fold to freshly isolated hepatocytes. The caspase-3 activities on days 6 and 7 were significantly higher than others (Figure 3a). Caspase-3 activation levels of 1 and 2 days cultured hepatocytes and of liver slices were similar.

Lower levels of caspase-3 activation in isolated hepatocytes corresponded to lack of caspase-9 activation. STS treatment could result in activation of caspase-9 in liver slices to 220% of untreated control, but not in the early days of hepatocytes culturing (Figure 3b). The caspase-9 activities of the STS-treated samples on days 0, 1 and 2 were similar to the untreated controls, while there was a small, but significant, increase on the day 3. Only on days 6 and 7 were the caspase-9 activities highly increased above the levels of untreated controls (by 4- and 5-fold, respectively). Even though caspase-9 was efficiently activated only in the liver slices and in the week-old hepatocytes, the overall difference between the samples was statistically significant (*P* = 9 × 10^−9^, Kruskal–Wallis rank sum test). In comparison to liver slices, caspase-9 activation significantly decreased in primary hepatocytes upon isolation and on days 1 and 2 after isolation. A small rise in caspase-9 activity on day 3 of culturing was not statistically significant when compared to the liver, nor to the activity on days 0, 1, 2 and 6. At this time point the inactivation of caspase-9 started to revert. This modest increase of caspase-9 activation was reflected in an increase of caspase-3 activity. The even larger increases of caspase-9 activities on days 6 and 7, surpassing those in the liver slices and in hepatocytes upon isolation by 2- and 6-fold, resulted in proportional increases of caspase-3 activity. The differences in activity of caspase-9 were statistically significant from that of the liver only on day 7.

Protein levels of the Mcl-1 and Bcl-xL, the two essential antiapoptotic proteins in hepatocytes, also differed during the hepatocyte cultivation (Figure 3c). The Mcl-1 amounts sharply increased on day 1, while a shorter form was additionally detected immediately after isolation and on days 3 and 6. The expression of Bcl-xL sharply increased on day 1 and remained similar to day 6. Faint larger bands of about 50 kDa were observed in liver and in primary hepatocytes immediately after isolation and on day 6; this antibody is certified as specific to Bcl-xL only [27].

Addition of NAC resulted in a large activation of caspase-3 and 9 (Figure 3d,e). These differences were statistically significant in the presence of NAC on the day of isolation. The antioxidant addition, therefore, can enable mitochondrial apoptosis triggering through the caspase-9 (*P* = 0.016, two-way ANOVA).

### 3.4. Urea Production in Isolated Primary Hepatocytes

**Figure 4:**
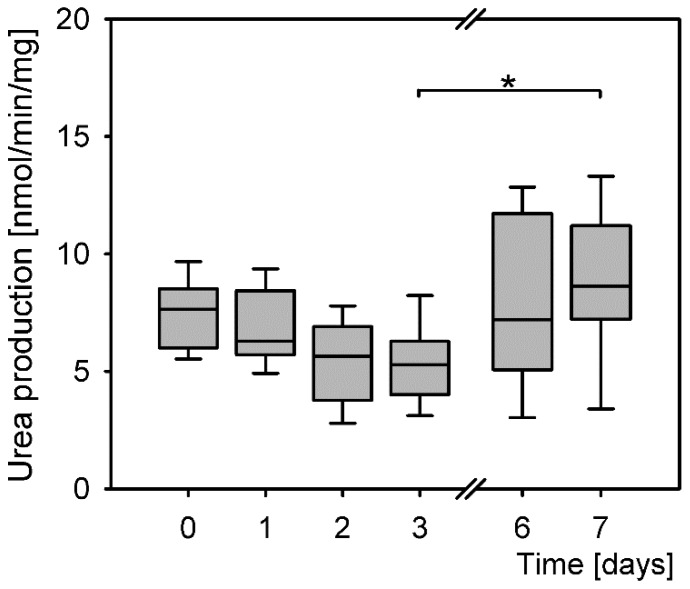
Differences in levels of urea production of primary hepatocytes (*P* = 0.002, Kruskal–Wallis rank sum test). Post hoc tests: days 3 and 7 (*P* = 0.039, Dunnett T3). *: *P* < 0.05.

Primary hepatocytes maintained their function from isolation throughout the culturing up to day 7 post isolation (Figure 4). The levels of produced urea were similar at all time points; however, the differences were statistically significant (*P* = 0.002, Kruskal–Wallis rank sum test). Further calculations of the probability of differences between the treatment groups revealed a significant difference only between the lowest and the highest levels of urea production on days 3 and 7.

## 4. Discussion

Isolation of primary hepatocytes is stressful, even if all parameters (media composition, temperature, pH, oxygenation, etc.) are ideally controlled. Cellular junctions, crucial for the normal function of hepatocytes and liver [28,29], must be disrupted by collagenase digestion. Collagenase activity and its effect on hepatocytes’ physiology can differ between collagenases from different sources and even different batches of the same enzyme [unpublished observation] and is a critical factor in hepatocytes’ isolation [30]. Therefore, there seems no way around the stress induction upon the hepatocyte isolation. Increased antioxidative response and H_2_O_2_ concentrations immediately after isolation support the existence of a stressor at the time of isolation. Larger H_2_O_2_ production and an increase in antioxidative enzymes activities were simultaneously detected reflecting the upregulation of an antioxidative response and a pro-survival strategy. Indeed, the majority of hepatocytes survived and no apoptotic morphology was observed. As hepatocytes survived more than 6 days after the ROS increase and retained their function, the increased H_2_O_2_ concentration clearly did not induce cell death.

Levels of H_2_O_2_ production were similar to those reported in the literature [4]. The differences in antioxidant enzyme activities and glutathione levels corresponded perfectly with one another: the enzyme activities increased upon the isolation, while the amount of total glutathione was slightly lower compared to that in liver and to that of hepatocytes in 3 days culture; however, this decrease was not statistically significant. Increased synthesis of glutathione on days 1 and 2 also reflected its increased utilization, as such compensations after its increased use were reported before [31]. The statistically significant increases or decreases of GPx and CAT activities occurred at exactly the same time points. Statistically, the same activities were in the liver, days 1 and 2 and then on days 6 and 7. Compared to the average activity of the liver, day 1 and day 2, the CAT activity seemed to increase more than that of GPx after the isolation (by 63% and 36%, respectively) and was reduced more on day 3 (by 37% and 25%, respectively). This CAT activity increase reflects that, unlike GPx, CAT is activated in the case of a larger production of H_2_O_2_ in hepatocytes [32]. The much lower activities of both enzymes on days six and seven may reflect cell aging. This is supported by a similar decrease of total glutathione at the same time point. SOD was significantly activated only upon the hepatocyte isolation pointing out to the increased amounts of superoxide. Therefore, at least these two ROS were formed during the acute stress. In contrast to the CAT and GPx activities, there was no sharp decrease of the SOD activity on days 6 and 7, indicating that continuous superoxide anion production had been compensated by SOD activity. SOD converts superoxide anion to H_2_O_2_, which can efficiently diffuse through cells and is considered to be a central redox signaling molecule [4]. The upregulation of antioxidative enzymes SOD, GPx and glutathione reductase for about 5 days was reported after liver transplantation [33].

Inability to activate caspase-9 (PACOS) is another stress response that was observed upon hepatocyte isolation. The expression of the two non-redundant antiapoptotic BCL-2 family proteins crucial for the hepatocytes’ integrity, Bcl-xL and Mcl-1, peaks 24 h after hepatocyte isolation. Additionally a shorter Mcl-1 variant is seen immediately after the isolation and disappears 24 h later. A Mcl-1 splicing variant, Mcl-1S, was described as proapoptotic [34]. These findings and inability to activate the caspase-9 are in line with previously published data [20,21]. An increase of the shorter Mcl-1 variant and increased amounts of Bcl-xL is expected on day 6, since the primary hepatocytes survive only for a short time in the culture with reducing number of cells, especially after day 5 [35]. Caspase-9 was partially activated from day 3 and recovered by day 6. This activation was reflected in increased levels of caspase-3 activation. Caspase-3 was active even in the absence of caspase-9 activity; however, it was activated even more when triggered by the caspase-9. This implies that there is another pathway to activate caspase-3, which is in line with the report that caspase-9 is dispensable for caspase-3 activation when apoptosis is triggered through the extrinsic pathway [15]. 

Increased amounts of H_2_O_2_ and superoxide were formed at hepatocyte’s isolation when cell junctions were disrupted. This is reminiscent of the increase in ROS production reported in detached endothelial cells [36]. There the increased ROS can trigger apoptosis. In hepatocytes, the function of moderately increased ROS results in stress adaptation to reduce apoptosis. Similar observations were reported in skeletal muscle cell models where ROS overproduction has different roles, depending on the magnitude, duration (acute versus chronic), origin of the ROS formation and cellular antioxidant status [37,38]. Excess ROS/RNS formation in skeletal muscle in response to sufficient intensity of physical exercise mediates favorable muscle adaptive responses by facilitating glucose uptake or inducing mitochondrial biogenesis and muscle hypertrophy. Conversely, after intense unaccustomed exercise oxidative stress accelerates age-dependent skeletal muscle wasting [39,40,41] and contributes to muscle cell dysfunction, inflammation and death [37,42,43,44,45,46,47]. 

**Figure 5:**
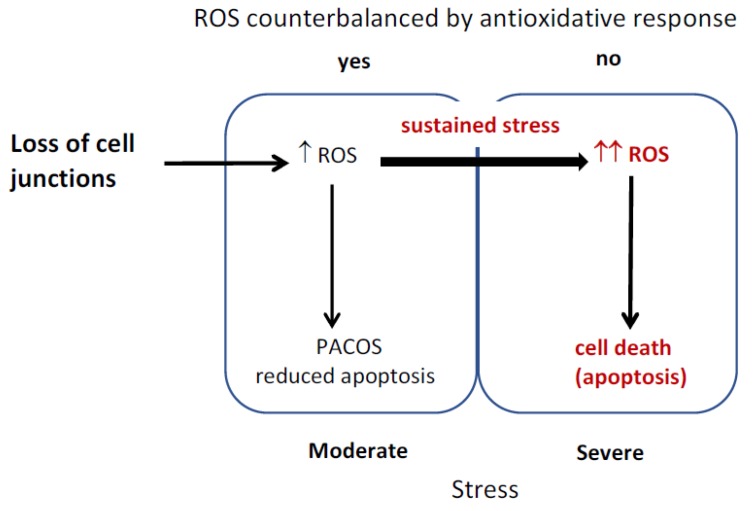
Reactive oxygen species (ROS) induction of stress responses and cell death.

Hepatocytes were reported to produce different amounts of ROS under stress, as human hepatocytes isolated from normal and diseased tissue differed in the ROS production under ischemia/reperfusion [7]. The diagram of the proposed model is depicted in Figure 5. Stress induced by loss of cell junctions is conveyed through ROS increase to trigger PACOS that prevents unnecessary apoptosis triggering in the presence of a mild stressor. ROS are counterbalanced by an antioxidative response, apoptosis is reduced and cell damage is repaired through adaptive stress responses. Sustained stress or excessive amounts of ROS result in cell death. The cells remain normal, when timely and efficient neutralization of ROS and induced stress responses prevent mutations that would otherwise result in malignant transformation of surviving cells.

## 5. Conclusions

The consequences of stress at hepatocytes’ isolation are two reversible stress responses: the induction of an endogenous antioxidative response and the reduced ability of apoptosis triggering, PACOS. Both of them are triggered by increased ROS and enable cell survival and normal function. 

Stress responses and ROS production occur and have a function *in vivo* in normal and diseased livers. ROS boost cell responses for increased survival, as they are needed for preconditioning in normal liver [10]. Excessive ROS production occurs in pathologies like alcoholic liver disease and others [8,48]. Oxidative imbalance also leads to disorders: the sustained low-grade oxidative stress is believed to promote progression to liver fibrosis and may increase risk for mid-term graft failure [11]. Transplanted tissue can encounter high ROS in the acceptor organism or in response to the transfer. Increased amounts of antioxidant enzymes were detected after transplantation [8,33]. In conclusion, understanding mechanisms of ROS signaling and stress responses is crucial for understanding liver physiology, pathophysiology and devising interventions for prevention and reversal of liver pathology.

## Abbreviations

CAT	catalase
GPx	glutathione peroxidases
H_2_O_2_	hydrogen peroxide
NAC	N-acetylcysteine
NADPH	nicotinamide adenine dinucleotide phosphate
PACOS	preapoptotic cell stress response
ROS	reactive oxygen species
SODs	superoxide dismutases
STS	staurosporine
